# Effects of Binahong (*Anredera cordifolia* (Tenore) Steenis) Extracts on the Levels of Malondialdehyde (MDA) in Cataract Goat Lenses

**DOI:** 10.1155/2021/6617292

**Published:** 2021-06-12

**Authors:** Feriyani Feriyani, Hady Maulanza, Rodiah Rahmawaty Lubis, Ummu Balqis, Darmawi Darmawi

**Affiliations:** ^1^Graduate School of Mathematics and Applied Sciences, Universitas Syiah Kuala, Banda Aceh, Aceh, Indonesia; ^2^Faculty of Medicines, Universitas Abulyatama, Aceh Besar, Aceh, Indonesia; ^3^Department of Ophthalmology, Faculty of Medicine, Universitas Sumatera Utara, Medan, Indonesia; ^4^Laboratory of Pathology, Faculty of Veterinary Medicine, Universitas Syiah Kuala, Banda Aceh, Aceh, Indonesia; ^5^Laboratory of Research, Faculty of Veterinary Medicine, Universitas Syiah Kuala, Banda Aceh, Aceh, Indonesia; ^6^Laboratory of Microbiology, Faculty of Veterinary Medicine, Universitas Syiah Kuala, Banda Aceh, Aceh, Indonesia

## Abstract

Cataracts are one of the most causes of blindness in the world. Oxidative stress can form pathological conditions such as cataracts. This oxidative stress ability can be measured by the malondialdehyde (MDA) biomarker. Binahong leaves (*Anredera cordifolia* (Tenore) Steenis) are native plants from Indonesia that are used to treat various diseases including cataract treatment. Binahong leaf (*Anredera cordifolia* (Tenore) Steenis) has a high amount of flavonoids and is rich in antioxidants that can be used to treat cataracts. *Objective*. The purpose of this study was to assess the effect of binahong leaf extract on the levels of MDA in a goat lens with cataract-induced material. *Method*. As many as possible, 40 goat eye lenses were divided into several groups, namely, group I normal lenses as controls (glucose 5.5 mM), group II lenses were cataract induced with glucose concentration of 55 mM, group III lenses with glucose 55 mM + binahong leaf extract (100 *μ*g/ml), group IV lens with glucose 55 mM + binahong leaf extract (200 *μ*g/ml), and group V lens with glucose 55 mM + quercetin (positive control). Biochemical parameters measured in the lens homogenate are malondialdehyde lens morphology in all groups' observations and comparisons made. *Results*. The results of the study found that the lens group with the addition of binahong extract showed more results transparency compared to lens groups induced by glucose concentrations of 55 mM). This shows that the diabetic cataract group experienced high oxidative stress due to the accumulation of sorbitol compounds derived from glucose which caused turbidity in the goat eye lens and increased levels of lens MDA. Binahong levels at concentrations of 100 or 200 can inhibit MDA production. *Conclusion*. Binahong (*Anredera cordifolia* (Tenore) Steenis) extract has the ability to inhibit the production of MDA levels. In glucose-induced goat lenses, binahong extract and quercetin show antioxidant and anticataract properties.

## 1. Introduction

A cataract is a condition of pacification of the eye lens that reduces the amount of light entering the eye, so that it can cause blindness [1]. The World Health Organization (WHO) suggests that by 2020, the number of blind people will reach 90 million globally [2, 3]. The prevalence of cataract increases with age, from 5% for patients aged 52–62 to 64% for patients over 70 years, in Europe [4]. The most common symptoms of cataract are impaired vision, decreased contrast sensitivity, color disturbance, and glare [5]. Cataracts can be caused by many factors such as oxidative stress, UV radiation, calcium levels in the lens, and complications of diabetes mellitus (diabetic cataracts) [6, 7]. Cataractogenesis in diabetes mellitus is mainly due to generation of free radicals causing oxidative stress. Oxidative stress is a condition where the amount of free radicals exceeds the amount of antioxidants found in the body. This condition can cause pathological conditions, one of which is cataract. This oxidative stress can also be defined as an increase in reactive oxygen species (ROS) and a physiological decrease in endogenous antioxidants [8] Oxidative stress is closely related to cataracts. Oxidative stress occurs due to an imbalance between oxidants and antioxidants. This oxidative stress ability can be measured by the malondialdehyde (MDA) biomarker [7]. Malondialdehyde is a by-product of mutagenic lipid peroxidase [9].

The correlation of MDA as a biomarker of ROS can cause lens turbidity; so at higher MDA, the lipid peroxidase production increases and causes turbidity [10]. Correlation of MDA with oxidative stress increases the MDA level, so that it increasingly indicates the presence of oxidative stress conditions [11]. Although the restoration of the vision in people with cataracts is conducted through surgery, the costs and risks remain an issue [12]. Binahong is a medicinal plant. Biological activity test on binahong plants mentioned that this plant is useful as antihyperlipidemic, anti-inflammatory, analgesic, and antipyretic [13].

Binahong leaves are traditionally used to treat various types of diseases including cataract treatment. Binahong contains antioxidants, so it can increase endogenous antioxidants which function to capture free radicals and suppress oxidative stress, so that lens turbidity can be prevented and can automatically prevent cataracts. The binahong plant (*Anredera cordifolia* (Tenore) Stennis) is one of the species of the Basellaceae family which is widely used in medicine in the field of human health and also as an antimicrobial plant pathogen [14]. Binahong leaves are known to contain oleanolic acid. Binahong leaf is an alternative source of antioxidants that can be developed further as a natural ingredient for cataract treatment.

Binahong leaves as one of the native plants from Indonesia that have been widely used for diabetes, which is known as the Dheng San Chi leaf in China; in Europe, it is known as the heartleaf Madeira vine leaf; and in South America, it is known as Madeira-vine [15]. The number of studies reveals the benefits of binahong, but not much research has been carried out to evaluate the effect of binahong leaf extracts on MDA levels. This study aims to determine the effect of binahong leaf extract on MDA levels in the cataract lens model.

## 2. Materials and Methods

### 2.1. Binahong Leaves Profile

*Anredera cordifolia* (Tenore) Steenis or better known as binahong, according to the Indonesian Herbal Pharmacopoeia Literature Supplement 2, comes from the Basellaceae tribe and is supported by the results of determination No. 2285/IPH.1.01/If.07/IX/2018 at the Herbarium Bogoriense Institute of Biology Research Center LIPI Cibinong, West Java. Spectrophotometry analysis or quantitative analysis based on binahong leaves containing vitexin with pharmacognostic parameters was carried out following the standards listed in the Indonesian Herb Pharmacopeia [16]. Other benefits of binahong leaves cure typhoid fever, gastritis, gout, inflammation of the intestine, swelling of the liver, and kidney disorders and also have a role in healing wounds [17]. Phytochemical compounds found in binahong leaves are alkaloids, flavonoids, saponins, and glycosides.

### 2.2. Preparation of Extract

Binahong leaves as much as 2 kg were cleaned-washed and drained. After dried (without direct sunlight), the leaves were cut into small pieces and macerated with 70% ethanol for 3 days. The extract was concentrated using a vacuum rotary evaporator until the gel was formed.

### 2.3. Lens Culture

Fresh goat eyeballs are collected from slaughterhouses. The eyeball is taken and stored in a coolbox temperature of 0–4°C to be taken to the laboratory. The lens is taken by extraction extracapsular and incubated in aqueous humor/solvent humor (NaCl 140 mM, KCl 5 mM, MgCl_2_ 2 mM, NaHCO_3_ 0.5 mM, NaH (PO_4_)_2_ 0.5 mM, CaCl_2_ 0.4 mM, and glucose 5.5 mM) at 0.5 mM glucose room temperature and pH 7.8 for 72 hours. Penicillin 32 mg and streptomycin 250 mg were added to the culture media to prevent bacterial contamination [18, 19].

### 2.4. Generation of Cataract

Glucose in a concentration of 55 mM is used to induce cataracts [18]. At high concentrations, glucose in the lens is metabolized through the sorbitol pathway and accumulation of polyols (sugar alcohol), causing excessive hydration and oxidative stress. This causes cataractogenesis.

Study drugs and groups design: a total of 30 lenses are divided into groups as follows (*n* = 6 in each category):

- Group I. normal lens (control (glucose 5.5 mM))
- Group II. glucose 55 mM
- Group III. glucose 55 mM + binahong leaf extract (100 *μ*g/ml)
- Group IV. glucose 55 mM + binahong leaf extract (200 *μ*g/ml)
- Group V. glucose 55 mM + quercetin

### 2.5. Lens Opacity Evaluation (Lens Morphology)

After 72 hours of the incubation process, the lens is observed to assess the lens's turbidity by placing the lens on paper with the posterior surface touching the paper for taking photographs and observing the visible box for evaluation of the lens's opacity [20]. To study the morphology of the lens, the lens is placed above the grid line, and then, the change in lens transparency assessed by taking into account the number and characteristics of the grid (grid line) is photographed.

The lens transparency degree score includes [21] 0, clear lens without turbidity (clearly visible grid line); 1, lens looks blurry and slightly visible degree of turbidity (looks a bit turbidity on grid line but grid line still appears); 2, lens appears blurry and diffuse pacification of the entire lens (moderate level turbidity on the grid line but still visible); and 3, lens appears opaque and thick turbidity across the lens (total turbidity with invisible grid line).

### 2.6. Homogenate Preparation

After incubating for 72 hours, a lens homogenate weighing 10% w/v was prepared in a Tris buffer solution (0.23 mM, pH 7.8) containing 0.25 × 10^−3^ M.

EDTA homogenates were centrifuged at a speed of 10,000 rpm for 1 hour, and a supernatant (lens homogenate) was used to estimate the biochemical examination parameters, namely, the determination of MDA.

### 2.7. Examination Parameters Estimation of Malondialdehyde (MDA)

Lipid peroxidation as evidenced by the formation of reactive substances thiobarbituric acid (TBARS) and hydroperoxides (HP) was measured by the Nieshus and Samuelsson method [22, 23]. About 0.1 ml of tissue homogenate (Tris HCl buffer, pH 7.4) is added to 2 ml (1 : 1: 1 ratio) of the TBA-TCA-HCl reagent (thiobarbituric acid 0.37%, 0.25 N HCl, and 15% TCA) and placed in a waterbath for 15 minutes, cooled, and centrifuged at 1000 g at room temperature for 10 minutes. Supernatant absorbance was measured against a blank solution at 535 nm. The values are expressed as MDA/min/mg lens protein polymers. Based on its ability as an antioxidant from the leaves of binahong, it can be assumed that binahong leaf plants can balance the imbalance that occurs in the lens.

## 3. Results and Discussion

### 3.1. Lens Morphology

**Figure 1:**
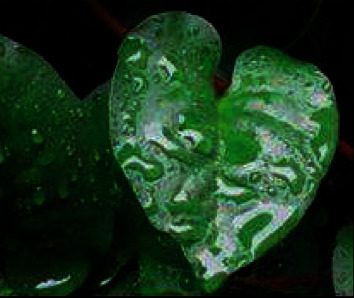
Binahong leaf (*Anredera cordifolia* (Tenore) Steenis).

Observation of lens turbidity after 72 hours in incubation is shown in Figure 1. In the description, (a) is a normal lens (clear lens without turbidity), (b) is a lens in glucose 55 mM (loss of transparency, no visible lines), (c) is a lens in glucose 55 mM + binahong extract 100 *μ*g/ml (lens looks blurry and diffuse opacification of the entire lens, but the grid line is still visible), (d) is a lens in glucose 55 mM + binahong extract 200 *μ*g/ml (lens looks blurry and slightly visible degree of turbidity), and (e) is a lens in glucose 55 mM + quercetin (lens appears opaque and slightly visible degree of turbidity). Oxidative stress is known to play a role in the development of diabetes and its complications such as cataracts. On exposure to high concentrations of glucose, the use of glucose in the lens begins through the sorbitol pathway. The accumulation of polyols (sugar alcohols) causes overhydration and oxidative stress resulting in the emergence of cataract generation [24]. Hyperglycemia induces oxidative stress through various pathways [25]. The crystalline oxidation of the lens as well as the membrane protein further results in the formation of insoluble protein aggregates. This study illustrates the loss of soluble protein from the lens due to conversion to insoluble protein due to lens protein oxidation as a reflection of the decrease in the total protein content of the lens.

### 3.2. Effect of Binahong Extract on MDA Malondialdehyde Levels on Cataract Lenses

Binahong leaf extract contains many secondary metabolites which have an antioxidant role. In Table 1, it can be seen that there are differences in the results between positive controls indicating that the value of quercetin actively inhibits turbidity from the lens. The binahong concentration results of 200 *μ*g approaching a value that approaches the positive control and is much lower than the negative control group (diabetic lens).

**Figure 2:**
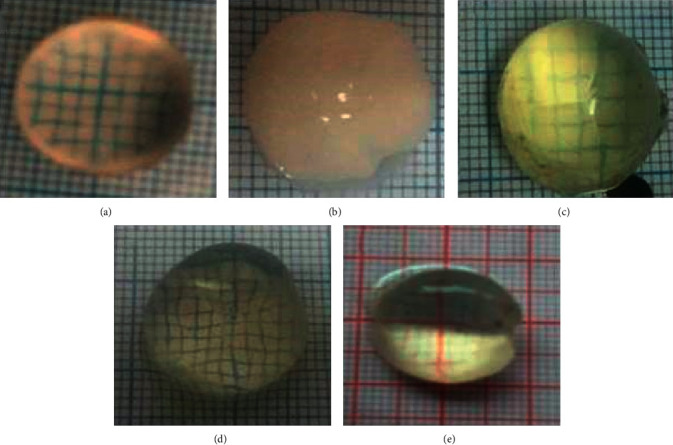
Comparison of lens turbidity. (a) Normal (grade 0). (b) Diabetic (grade 4). (c) Binahong 100 *μ*g/ml (grade 2). (d) Binahong 200 *μ*g/ml (grade 1). (e) Quercetin (grade 1).

Table 1 and Figure 2 show that binahong concentrations of both 100 and 200 have been able to inhibit MDA production. With doses of 100 and 200, it has shown a significant response. The decrease in MDA levels is thought to occur due to the high antioxidant content of this binahong leaf extract. Exogenous antioxidant sources from the binahong plant can be seen from the levels of vitamin C, vitamin E, carotenoids, and antioxidant enzymes such as superoxide dismutase (SOD), catalase (CAT), and (Se-GPx) [25]. The content of binahong metabolites contains antioxidants. The high binahong extract can be seen from the low levels of MDA from the eye lens. Low MDA levels also indicate that antioxidants can have anticataractogenesis activity. The antioxidant activity of binahong is indicated by the decreased levels of MDA as a biomarker of oxidative stress. There are several markers of oxidative stress, namely, MDA and lipid hydroperoxide. Antioxidants can act as a tool that will prevent the formation of ROS. Prevention of the formation of ROS in antioxidants act as a scavenger, trapping and eliminating ROS or acting as ions that bind metal ions into inactive cheating [26, 27]. MDA is known as a dominant damage product from lipid peroxide. In cortical cataracts in humans as well as in nuclear cataracts, MDA has been found in various studies to increase 3.5 times the normal level and identify a reactive substance 2-thiobarbiturate acid (TBARS) [28, 29]. The increased level of MDA in the experimental cataract model developed in this study mimics this effect seen in humans. Disruption of the defense mechanism against reactive oxygen metabolites has been shown in the lens of human cataracts which is expressed from a significantly decreased activity of various antioxidant enzymes [28].

**Figure 3:**
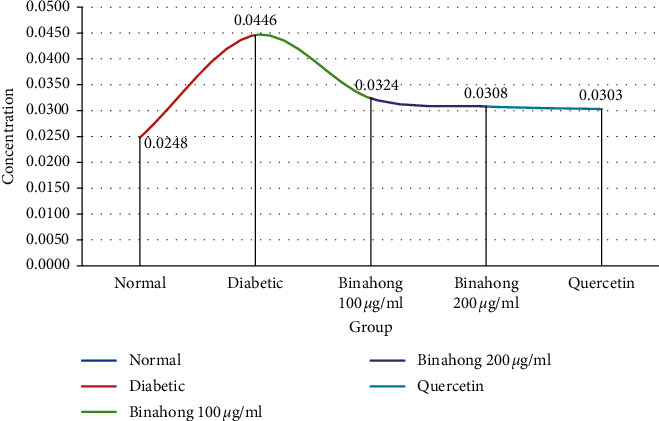
Effects of Binahong leaf extract on MDA level in the lens homogenate after 72 hours of incubation.

Figure 3 shows that there are 5 groups of lenses that have been homogenate after incubation for 72 hours, and their MDA levels were measured. In the normal group (negative control) and the diabetes group (positive control), MDA levels were 0.0248 *μ*g/g and 0.0466 *μ*g/g, whereas in the quercetin group (standard drug group) which was the lens group with diabetes plus quercetin, MDA is 0.0303 *μ*g/g. The diabetic lens group added with binahong leaf extract at a concentration of 100 *μ*g/ml and 200 *μ*g/ml obtained MDA levels of 0.0324 *μ*g/g and 0.0308 *μ*g/g comparison of the diabetes group with the binahong 100 and 200 groups, and there is an effect of reducing MDA levels in the binahong 100 and binahong 200 groups. Comparing the diabetes group to the quercetin group (standard drug group), the result is that there is an effect of reducing MDA levels in the quercetin group. These results indicate that there is an increase in MDA levels in the diabetic lens. A decrease in MDA levels occurred in lenses given binahong levels of 100 and 200. The decrease in MDA levels is thought to occur due to the high antioxidant content of binahong leaf extract. In addition to the presence of these antioxidants, secondary metabolites such as flavonoids are also able to inhibit cataractogenesis. Antioxidants are known to delay cataractogenesis [30]. Previous reports suggest that phytochemicals or natural plant products retard the process of cataractogenesis by scavenging free oxygen radicals. High antioxidant content can inhibit the process of cataractogenesis in the lens of the eye [31]. Based on the ability as an antioxidant from the leaves of binahong, it can be assumed that binahong leaf plants can balance the imbalance that occurs in the lens of cataract sufferers. Keratogenesis inhibition pathways that occur are through radical inhibitory pathways free by substituting phytol with reactive oxygen species (ROS). This antioxidant will change the oxidative atmosphere into oxidation. Flavonoids have been shown to increase the activity of antioxidant enzymes (superoxide dismutase and catalase) which can then prevent cataractogenesis induced by the next selenite [32, 33]. Plant extracts were more effective in preventing cataractogenesis than anthocyanidins, flavon-3ol, flavanone, total flavone, or total flavonol [34]. The majority of the anticataract compounds tested, including plant extracts and naturally occurring compounds, lies in their antioxidant and/or free radical scavenging and/or anti-inflammatory propensity [35].

## 4. Conclusions

Binahong (*Anredera cordifolia* (Tenore) Steenis) extract has the ability to inhibit the production of malondialdehyde (MDA) levels. The decrease in MDA levels is thought to occur due to the high antioxidant content of binahong leaf extract. In experimental trials of goat lens induction with glucose which produces diabetic cataracts, binahong leaf extract shows antioxidant properties by inhibiting the production of MDA. Binahong extract concentration at 100 *μ*g/ml with 200 *μ*g/ml shows that the binahong leaf extract has the anticataractogenesis activity.

## Figures and Tables

**Table 1:** Effect of binahong extract on MDA levels on cataract lenses.

Content of malondialdehyde (MDA)					
Subject	Normal	Diabetic	Binahong 100 *μ*g/ml	Binahong 200 *μ*g/ml	Quercetin
L1	0.0242	0.0563	0.0346	0.0308	0.0275
L2	0.0243	0.0500	0.0346	0.0308	0.0269
L3	0.0244	0.0376	0.0345	0.0308	0.0300
L4	0.0246	0.0563	0.0349	0.0308	0.032
L5	0.026	0.023	0.023	0.0309	0.036
*x* ± SD	0.0248 ± 0.001	0.0446 ± 0.014	0.0324 ± 0.005	0.0308 ± 0.000	0.0303 ± 0.004

## Data Availability

The data used to support the findings of this study are available from the corresponding author upon request.
